# ADM-Assisted Breast Reconstruction vs Micro-Polyurethane Foam-Covered Implants in the Prepectoral Space: A Monocentric Study

**DOI:** 10.1007/s00266-026-05675-9

**Published:** 2026-04-20

**Authors:** Sergio Miranda, Luca Mazzocconi, Giulio Tarantino, Samuele Frassoni, Vincenzo Bagnardi, Gaia Ghiringhelli, Pietro Caldarella, Francesca De Lorenzi

**Affiliations:** 1 Department of Plastic and Reconstructive Surgery, European Institute of Oncology, IRCCS, Milan, Italy; 2 University of Milan, Milan, Italy; 3 Department of Plastic, Reconstructive and Esthetic Surgery, Department of Science and Health of Women, Children and Public Health, Fondazione Policlinico Universitario Agostino Gemelli IRCCS, Rome, Italy; 4 GROW – School for Oncology and Developmental Biology, Maastricht University, Maastricht, The Netherlands; 5 Department of Statistics and Quantitative Methods, University of Milano-Bicocca, Milan, Italy; 6 Department of Breast Surgery, European Institute of Oncology, IRCCS, Milan, Italy

**Keywords:** Prepectoral breast reconstruction, Direct-to-implant (DTI), Micro-polyurethane foam-coated implants, ADM, Breast cancer

## Abstract

**Background:**

Prepectoral breast reconstruction using acellular dermal matrices (ADMs) or micro-polyurethane foam-covered breast implants have emerged as commonly used surgical techniques. Although the use of both ADMs and micro-polyurethane foam-covered implants in prepectoral breast reconstruction are widely described in literature, comparative data remains limited.

**Objectives:**

Our goal was to compare the short-term and medium-term clinical outcomes as well as the patient-reported outcomes in patients undergoing direct-to-implant (DTI) prepectoral breast reconstruction using ADM versus micro-polyurethane foam-covered implants.

**Methods:**

A retrospective matched cohort study was conducted on 64 patients (32 ADM-wrapped and 32 micro-polyurethane foam-covered breast implants). Patients were matched based on prior surgery, adjuvant radiotherapy, and implant volume. Demographic and oncological characteristics, surgical variables, short- and medium-term outcomes, and patient-reported outcomes were compared between the two groups.

**Results:**

The micro-polyurethane group had significantly older patients (median age 53 vs. 47 years; *p* = 0.036). Periprosthetic fluid collection (31% vs. 3%; *p *= 0.006) and need for percutaneous fluid aspiration (38% vs. 9%; *p *= 0.016) occurred significantly more often in the ADM group. The indication for further surgery was also higher with ADM (47% vs. 19%; *p *= 0.031). Patient satisfaction showed a non-significant trend favoring micro-polyurethane implants in satisfaction with outcome domain (mean: 89 vs. 82; *p *= 0.060).

**Conclusions:**

Both ADM-assisted reconstructions and micro-polyurethane implants demonstrated high performance in the short and medium term, and they were associated to high patient satisfaction with reconstruction. However, micro-polyurethane implants were associated with fewer periprosthetic fluid collections and a lower need for percutaneous fluid aspirations compared to the ADM group. These findings suggest a potential advantage in terms of reduced postoperative complications.

**Level of Evidence III:**

This journal requires that authors assign a level of evidence to each article. For a full description of these Evidence-Based Medicine ratings, please refer to the Table of Contents or the online Instructions to Authors www.springer.com/00266.

## Introduction

Breast reconstruction has evolved dramatically over recent decades, driven by advances in mastectomy techniques and a growing emphasis on patient outcomes and esthetics [1–3]. Among the most significant innovations has been the renewed interest in prepectoral reconstruction. Compared to traditional subpectoral implants, prepectoral ones avoid pectoralis muscle manipulation, reducing postoperative pain, preserving upper body strength, and preventing animation deformity [4–7].

The widespread adoption of the prepectoral technique has been supported by two principles: the use of acellular dermal matrices (ADMs) and micro-polyurethane foam-coated implants [8–11]. ADMs are derived from decellularized human or animal dermis and primarily consist of collagen and glycosaminoglycans that support neovascularization, fibroblast infiltration, and integration [11]. In reconstructive surgery, ADMs serve to stabilize the implant, reinforce mastectomy flaps, and reduce capsular contracture by modulating local inflammatory responses [11].

In contrast, micro-polyurethane implants offer distinct mechanical and biological benefits. They consist of silicone gel prostheses covered with a microporous polyurethane foam that modulates fibroblast activity and collagen organization at the implant-tissue interface [12, 13]. The polyurethane (PU) coating adheres rapidly to surrounding tissues, creating a "Velcro-like" interface that enhances implant stability and minimizes dead space [14]. Over time, the PU foam undergoes gradual surface breakdown while being replaced by a stable fibrovascular capsule at the tissue–implant interface. Additionally, the unique surface texture alters fibroblast behavior and collagen deposition, contributing to a significantly lower incidence of capsular contracture over time [3, 15–18].

Clinical comparisons between ADM-assisted reconstructions and micro-polyurethane implants are limited but growing. Early reports suggest that polyurethane implants may be associated with fewer early complications, shorter operative times, and lower overall costs without compromising esthetic or patient-reported outcomes [15]. Historical concerns over degradation byproducts of polyurethane foam have been largely addressed by long-term studies demonstrating their safety and biocompatibility [19–21].

Despite their rising popularity, head-to-head studies comparing these two techniques remain sparse, particularly within the context of prepectoral reconstruction. Given the distinct mechanical behavior and tissue interactions of ADMs and polyurethane implants, understanding their complication profiles and effects on patient satisfaction is essential for optimizing indications.

The objective of this matched cohort study is to compare the short-term and medium-term clinical outcomes and patient-reported outcomes of women undergoing prepectoral reconstruction with either ADMs or micro-polyurethane implants.

## Materials and Methods

### Study Design

A monocentric retrospective matched cohort study was conducted at the European Institute of Oncology, Milan, Italy. Institutional Review Board approval was obtained (UID4322), and all aspects of this study were conducted in accordance with the Declaration of Helsinki. Since 2019, all patients who underwent immediate postmastectomy prepectoral reconstruction were recorded in an institutional database.

Eligible patients included those who underwent mastectomy and immediate prepectoral reconstruction. We excluded all patients who underwent bilateral mastectomy, active smokers, major comorbidities, history of previous breast irradiation, skin reducing pattern mastectomies, and hybrid techniques with fat grafting.

One group was composed of patients who underwent mastectomy and prepectoral reconstruction with anatomical breast implants and Braxon®, an ADM derived from porcine dermis. Braxon® is 0,6 mm thick, natural, sterilized with ethylene oxide to preserve collagen’s native structure, and shaped for complete implant wrapping.

The other group was composed of patients who underwent mastectomy and prepectoral reconstruction with micro-polyurethane implants, without the use of additional ADMs or meshes.

The micro-polyurethane group was used to find a control for each ADM patient, matching for previous surgery, adjuvant radiotherapy, and implant volume (±80 cc).

All procedures were performed using a standardized approach. In the ADM group, anatomically shaped silicone gel implants were fully wrapped in a porcine-derived ADM (Braxon®), placed in the prepectoral plane, and sutured to the underlying pectoral fascia and overlying subcutaneous tissue to reduce dead spaces. In the micro-polyurethane group, the micro-polyurethane foam-covered implants were placed in the prepectoral pocket without ADM. A closed suction drain was placed in all cases.

Data collection included demographic, oncologic, and surgical variables for each patient. All patients included in the study underwent routine follow-up visits performed at one week, one month, 12 months, and subsequently every year.

Short-term outcomes within 90 days from surgery and included time to drain removal (in days), periprosthetic fluid collections, hematoma, surgical site infection, flap necrosis, and implant explantation. Periprosthetic fluid collection was defined as fluid accumulation around the implant > 40 mL, diagnosed clinically or with ultrasound. The cutoff value of 40 mL was intentionally conservative to capture even minimal fluid accumulations with potential clinical relevance.

Medium-term outcomes, within 12 months from surgery, included capsular contracture, according to Baker classification, implant rippling, and the need for revisional surgery.

Patient satisfaction and quality of life were evaluated using the postoperative BREAST-Q™ Reconstruction Module (version 1.0), which assessed four domains: satisfaction with breasts, satisfaction with outcome, psychosocial well-being, and sexual well-being. Responses were scored on a 0–100 scale, with higher scores reflecting greater satisfaction or well-being (Table 1).

**Table 1:** BREAST-Q TM Version 1.0 - post-operative reconstruction module

Satisfaction with breasts	Satisfaction with outcome
This 15-item scale measures body image in terms of a woman’s satisfaction with her breasts and asks questions regarding how comfortably bras fit and how satisfied a woman is with her breast area both clothed and unclothed. The possible answers are: Very Dissatisfied, Somewhat Dissatisfied, Somewhat Satisfied, Very Satisfied.	This 7-item scale measures a woman’s overall appraisal of the outcome of her breast surgery. The possible answers are: Disagree, Somewhat Agree, Definitely Agree.
How you look in the mirror clothed?	Having reconstruction is much better than the alternative of having no breast(s).
The shape of your reconstructed breast(s) when you are wearing a bra?	I would encourage other women in my situation to have breast reconstruction surgery.
How normal you feel in your clothes?	I would do it again.
The size of your reconstructed breast(s)?	I have no regrets about having the surgery.
Being able to wear clothing that is more fitted?	Having this surgery changed my life for the better.
How are your breasts lined up in relation to each other?	The outcome perfectly matched my expectations.
How comfortably your bras fit?	It turned out exactly as I had planned.
The softness of your reconstructed breast(s)?	
How equal in size your breasts are to each other?	
How natural your reconstructed breast(s) looks?	
How naturally your reconstructed breast(s) sits/hangs?	
How your reconstructed breast(s) feels to touch?	
How much your reconstructed breast(s) feels like a natural part of your body?	
How closely matched your breasts are to each other?	
How you look in the mirror unclothed?	

Psychosocial well-being	Sexual well-being
This 10-item scale measures psychosocial well-being with items that ask about body image (e.g., accepting of body, feeling attractive) and a woman’s confidence in social settings. The possible answers are: None of the time, A little of the time, Some of the time, Most of the time, All of the time.	This 6-item scale measures sexual well-being with items that ask about feelings of sexual attractiveness (clothed and unclothed), sexual confidence as it relates to one’s breasts and feeling comfortable or at ease during sexual activity. The possible answers are: None of the time, A little of the time, Some of the time, Most of the time, All of the time.
Confident in a social setting?	Sexually attractive in your clothes?
Emotionally able to do the things that you want to do?	Comfortable/at ease during sexual activity?
Emotionally healthy?	Confident sexually?
Of equal worth to other women?	Satisfied with your sex-life?
Self-confident?	Confident sexually about how your breast(s) look when unclothed?
Feminine in your clothes?	Sexually attractive when unclothed?
Accepting of your body?	
Normal?	
Like other women?	
Attractive?	

Continuous variables were summarized as medians and ranges or as mean and standard deviation (SD). Categorical variables were presented as counts and percentages. Comparisons between the two matched groups were performed using Wilcoxon signed-rank test for continuous variables and Fisher’s exact test for categorical variables. All reported p-values were two-sided, with p-values less than 0.05 considered statistically significant. Statistical analyses were conducted using SAS software, version 9.4 (SAS Institute, Cary, NC, USA).

## Results

As of December 2022, 287 patients underwent mastectomy and immediate prepectoral reconstruction with ADM (*n* = 43) or micro-polyurethane implants (*n* = 244). We included all reconstructions performed between March 2020 and July 2022, therefore excluding from this series our early experience with the prepectoral technique and patients with shorter follow-up. In this interval, we identified 33 patients who underwent ADM-assisted reconstructions. One further patient was excluded since she underwent a bilateral mastectomy in the same interval. So far, we retrospectively identified a matched cohort of 64 patients who underwent mastectomy and prepectoral reconstruction: 32 patients with micro-polyurethane were matched to the 32 ADM-wrapped patients, according to previous surgery on the affected breast, adjuvant radiotherapy, and implant volume. The median follow-up was 20 months (interquartile range: 13-26 months).

### Demographic and Oncologic Characteristics

In the matched cohort, baseline demographic and oncologic characteristics were well balanced between the two groups, except age (Table 2). Patients in the polyurethane group were significantly older, with a median age of 53 years compared to 47 years in the ADM group (*p* = 0.036). Micro-polyurethane patients also had a higher median BMI (22.6 vs. 21.5, *p* = 0.067). No significant differences were observed in smoking status, tumor histology, grade, stage, or in terms of adjuvant or neoadjuvant therapies.

**Table 2:** Demographic and oncologic characteristics of patients in the ADM and micro-polyurethane groups

		ADM (*N* = 32)	Micro-polyurethane (*N* = 32)	*P*-value
Age, median (min–max)		47 (30–67)	53 (35–69)	0.036
BMI, median (min–max)		21.5 (18.0–27.0)	22.6 (18.3–29.8)	0.067
Tobacco use, *N* (%)	No	32 (100)	31 (97)	1.00
	Yes	0 (0)	1 (3)	
Previous surgery, *N* (%)	No	29 (91)	29 (91)	*
	Yes	3 (9)	3 (9)	
Neoadjuvant radiotherapy, *N* (%)	No	32 (100)	32 (100)	1.00
	Yes	0 (0)	0 (0)	
Neoadjuvant chemotherapy, *N* (%)	No	30 (94)	30 (94)	1.00
	Yes	2 (6)	2 (6)	
Histology, *N* (%)	Invasive ductal carcinoma	20 (63)	17 (53)	0.78
	Invasive lobular carcinoma	4 (13)	6 (19)	
	DCIS	8 (25)	8 (25)	
	Fibrocystic disease	0 (0)	1 (3)	
G, *N* (%)	0	0 (0)	1 (3)	0.97
	1	6 (23)	5 (17)	
	2	12 (46)	15 (50)	
	3	8 (31)	9 (30)	
	Missing	6	2	
pT, *N* (%)	pT0	0 (0)	1 (3)	1.00
	pT1	18 (56)	18 (56)	
	pT2	5 (16)	6 (19)	
	pT3	2 (6)	1 (3)	
	pTis	7 (22)	6 (19)	
pN, *N* (%)	pN0	25 (78)	26 (81)	1.00
	pN1	5 (16)	5 (16)	
	pN2	1 (3)	1 (3)	
	pNx	1 (3)	0 (0)	
Adjuvant chemotherapy, *N* (%)	No	19 (59)	25 (78)	0.18
	Yes	13 (41)	7 (22)	
Adjuvant radiotherapy, *N* (%)	No	28 (88)	28 (88)	*
	Yes	4 (13)	4 (13)	
Hormonal therapy, *N* (%)	No	9 (28)	13 (41)	0.43
	Yes	23 (72)	19 (59)	
Monoclonal antibodies, *N* (%)	No	29 (91)	27 (84)	0.71
	Yes	3 (9)	5 (16)	

*Matching variables

### Surgical Variables

Surgical variables were comparable between the two groups and treated over the same period. Median implant volume was 335 mL in the ADM group and 330 mL in the micro-polyurethane group. Axillary dissection was performed in 2 patients (6%) in the ADM group and 5 patients (16%) in the micro-polyurethane group (*p* = 0.43). Nipple-areola sparing mastectomy was performed in all ADM patients (100%) and 30 patients (94%) in the micro-polyurethane group (*p* = 0.49). Surgery of the contralateral healthy breast was performed in 13 patients (41%) in the ADM group and 8 patients (25%) in the micro-polyurethane group (*p* = 0,29). The surgical variables are summarized in Table 3.

**Table 3:** Surgical variables of patients in the ADM and micro-polyurethane groups

		ADM (*N* = 32)	Micro-polyurethane (*N* = 32)	*P*-value
Year of surgery, *N* (%)	2020	10 (31)	6 (19)	0.14
	2021	12 (38)	20 (63)	
	2022	10 (31)	6 (19)	
Sentinel node biopsy, *N* (%)	No	1 (3)	1 (3)	1.00
	Yes	31 (97)	31 (97)	
Axillary dissection, *N* (%)	No	30 (94)	27 (84)	0.43
	Yes	2 (6)	5 (16)	
Nipple areola sparing, *N* (%)	No	0 (0)	2 (6)	0.49
	Yes	32 (100)	30 (94)	
Intent for mastectomy, *N* (%)	Therapeutic intent	32 (100)	31 (97)	1.00
	Prophylactic intent	0 (0)	1 (3)	
Implant volume, median (min–max)		335 (180–615)	330 (195–615)	*
Contralateral surgery, *N* (%)	No	19 (59)	24 (75)	0.29
	Yes	13 (41)	8 (25)	

*Matching variable

### Short-Term Outcomes

Short-term outcomes revealed significant differences in periprosthetic fluid collection that occurred in 31% of patients in the ADM group compared to 3% in the polyurethane group (*p* = 0.006). Among the 10 patients in the ADM group with periprosthetic fluid collection, the median quantity of fluid was 80 mL (range: 40–200 mL).

The need for percutaneous aspiration was significantly higher in the ADM group (38% vs. 9%, *p* = 0.016). Although not statistically significant, the time to drain removal trended longer in the ADM group (median 10 vs. 8 days, *p* = 0.074).

Postoperative bleeding occurred in 1 patient (3%) of the ADM group and did not occur in the micro-polyurethane group. Implant infection and subsequent explantation occurred in 2 patients (6%) in the ADM group and 1 patient (3%) in the micro-polyurethane group. Skin necrosis of the mastectomy flaps occurred in 4 patients (13%) in the ADM group and 1 patient (3%) in the micro-polyurethane group.

The short-term outcomes are summarized in Table 4.

**Table 4:** Short-term postoperative outcomes in the ADM and micro-polyurethane groups

		ADM (*N* = 32)	Micro-polyurethane (*N* = 32)	*P*-value
Time to drain removal (days), median (min–max)		10 (7–20)	8 (6–20)	0.074
Missing		0	2	
Periprosthetic fluid collection, *N* (%)	No	22 (69)	31 (97)	0.006
	Yes	10 (31)	1 (3)	
Percutaneous fluid aspiration, *N* (%)	No	20 (63)	29 (91)	0.016
	Yes	12 (38)	3 (9)	
Bleeding/Hematoma, *N* (%)	No	31 (97)	32 (100)	1.00
	Yes	1 (3)	0 (0)	
Infection, *N* (%)	No	30 (94)	31 (97)	1.00
	Yes	2 (6)	1 (3)	
Necrosis, *N* (%)	No	28 (88)	31 (97)	0.35
	Yes	4 (13)	1 (3)	
Explantation, *N* (%)	No	30 (94)	31 (97)	1.00
	Yes	2 (6)	1 (3)	

### Medium-Term Outcomes

Medium-term outcomes were assessed in 61 patients (30 in the ADM group and 31 in the polyurethane group), excluding those who underwent implant removal. A significantly higher proportion of patients in the ADM group required further surgical interventions (47% vs. 19%, *p* = 0.031), most of which were minor revisional procedures, such as lipofilling. The incidence of capsular contracture (Baker grade ≥ III) was low and not significantly different (4% ADM vs. 8% polyurethane, *p* = 0.59). Rippling or soft tissue irregularities were more frequent in the polyurethane group (64% vs. 47%); this difference did not reach statistical significance (*p* = 0.28).

Additional surgery occurred in 14 patients (47%) in the ADM group, of which nine patients underwent lipofilling. Six patients (19%) in the micro-polyurethane group underwent additional surgery, of which six were lipofilling.

The medium-term outcomes are summarized in Table 5.

**Table 5:** Medium-term outcomes in patients without implant explantation in the ADM and micro-polyurethane groups

		ADM (*N* = 30)	Micro-polyurethane (*N* = 31)	*P*-value
BAKER, *N* (%)	2	27 (96)	22 (92)	0.59
	3	1 (4)	2 (8)	
	Missing	2	7	
Rippling/soft tissue defects, *N* (%)	No	16 (53)	9 (36)	0.28
	Yes	14 (47)	16 (64)	
	Missing	0	6	
Indication for further surgery, *N* (%)	No	16 (53)	25 (81)	0.031
	Yes	14 (47)	6 (19)	
	Lipofilling	9	6	
	Other	2	0	
	Missing	3	0	

*.*

### Patient-Reported Outcomes

Patient-reported outcomes showed no statistically significant differences across domains. Satisfaction with breasts had a median score of 58 in the ADM group and 61 in the polyurethane group (*p* = 0.16). Although not statistically significant, satisfaction with outcome was higher in the polyurethane group, with a median score of 100 compared to 76 in the ADM group (*p* = 0.060). Psychosocial and sexual well-being scores were similar between groups, with median values of 70 and 59 in the ADM group, and 70 and 57 in the polyurethane group, respectively.

**Fig. 1:**
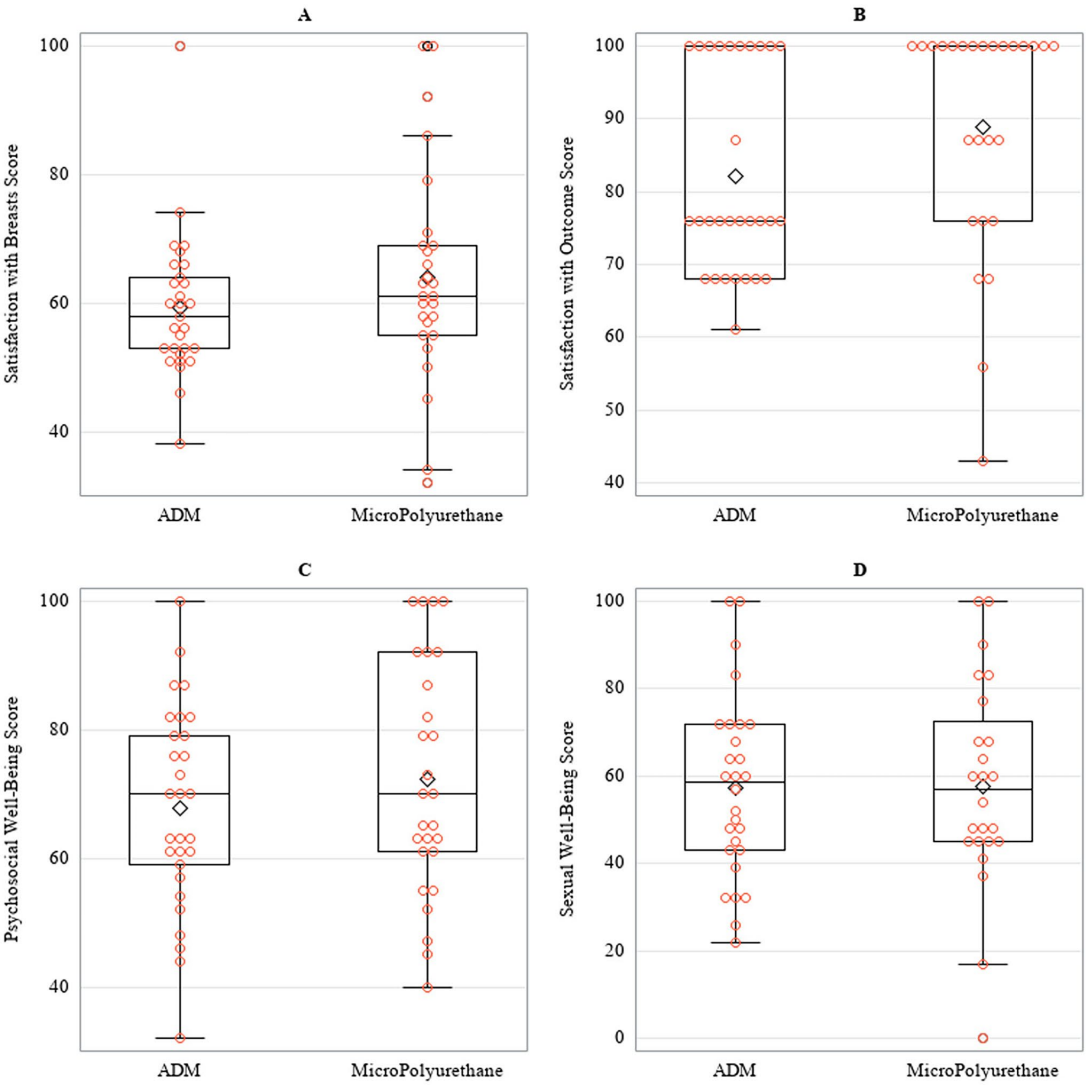
Median BREAST-Q scores across the four subscales (satisfaction with breasts, satisfaction with outcome, psychosocial well-being, and sexual well-being) in the ADM and micro-polyurethane groups.

The patient-reported outcomes are summarized in Table 6 and Fig. 1.

**Table 6:** Patient-reported outcomes assessed with the BREAST-Q™ questionnaire in the ADM and micro-polyurethane groups

	ADM (*N* = 32)	Micro-polyurethane (*N* = 32)	*P*-value
Satisfaction with breasts, median (min-max), mean (SD)	58 (38–100), 59 (11)	61 (32–100), 64 (17)	0.16
Missing	3	5	
Satisfaction with outcome, median (min-max), mean (SD)	76 (61–100), 82 (14)	100 (43–100), 89 (16)	0.060
Missing	3	6	
Psychosocial well-being, median (min-max), mean (SD)	70 (32–100), 68 (16)	70 (40–100), 72 (18)	0.39
Missing	3	5	
Sexual well-being, median (min–max), mean (SD)	59 (22–100), 57 (21)	57 (0–100), 58 (24)	0.88
Missing	4	8

## Discussion

Nowadays, prepectoral breast reconstruction is our first choice, when applicable. By placing the implant above the muscle, this method prevents animation deformity, allowing a quicker recovery, less postoperative discomfort and improved cosmetic outcomes when compared to the submuscular approach [7, 8, 18, 22, 23].

Nonetheless, selecting candidates and thoroughly evaluating flap perfusion is essential to ensure optimal and safe outcomes [3]. In addition, the use of ADM in covering prepectoral breast implants has demonstrated good esthetic results, with a low rate of postoperative complications [24]. However, the ADM cost, the volume and duration of surgical drainage output, and the reported risk of periprosthetic fluid collection are considered as weak points for this reconstructive technique [25].

In this matched cohort study, we compared prepectoral reconstruction using two distinct strategies: ADM-assisted reconstructions and micro-polyurethane implants. Patients were matched by implant volume (±80 cc), previous ipsilateral breast surgery, and adjuvant radiotherapy. These matching criteria were chosen to control key factors associated to postoperative complications and outcomes. Larger implants are associated to larger skin envelopes after mastectomy, potential perfusion compromise of the mastectomy flaps and possible increased tension, justifying volume-based matching. On the other hand, previous breast surgery and scarring impairs mastectomy flap perfusion and may increase the risk of complications. Adjuvant radiotherapy negatively influences capsular contracture rate. Although the year of surgery was not a formal matching variable, both groups were treated over the same period, ensuring balanced follow-up duration.

Periprosthetic fluid collection is reported as one of the most frequent complications and may result in infection and implant failure [26]. Its biology is multifactorial: flap thickness/perfusion, use of surgical drains, extent of surgery, and patient-specific factors. Some series have observed higher fluid collections with porcine ADM than with human allografts, although findings are heterogeneous and confounded by selection and technique. In our matched cohort, periprosthetic fluid collection and aspiration were significantly more frequent with ADM than with polyurethane (31% vs 3% and 38% vs 9%, respectively), aligning with reports that polyurethane’s early adhesion may reduce dead space. We used a conservative definition of periprosthetic fluid collection with a threshold value of 40 mL, diagnosed either clinically or with ultrasound. We arbitrarily registered a low cutoff value to avoid issues with implant or ADM integration. This approach likely contributed to the relatively high rate observed, which is difficult to compare to other studies, as there is no universal consensus about its definition whether human-derived ADM would attenuate this difference warrants study.

Correia-Pinto et al. similarly reported lower rates of periprosthetic fluid collection and infection with polyurethane implants compared to ADMs, reinforcing the mechanical and biological advantages of polyurethane surfaces [15, 27]. Caputo et al. also explored the relationship between ADM and periprosthetic fluid collection, reporting an increased incidence possibly due to the ADM’s hydrophilic nature [28]. Conversely, the microporous structure of polyurethane foam rapidly adheres to adjacent tissues through a "Velcro-like" effect, minimizing shear forces and fluid accumulation [7, 8, 22, 29, 30]. Our results suggest that the use of micro-polyurethane-coated implants is associated with a significantly lower rate of short-term fluid-related complications.

To further reduce periprosthetic fluid collection, careful surgical techniques and appropriate patient selection are essential. We integrated strategies such as limiting the use of electrocautery or using it on blend mode, preferring cold blade dissection when feasible, and avoiding antiseptics such as povidone-iodine with ADM, as it may impair fibroblast activity.

Similarly to previous literature, the iBAG study, the largest multicenter audit on ADM-assisted prepectoral reconstructions, emphasized that ideal candidates are non-smokers with good flap quality and moderate mastectomy weights and reported a low capsular contracture rate (2.1%) and implant loss (6.5%) in a real-world European population [31].

The choice of reconstructive strategy following mastectomy remains at the discretion of the plastic surgeon, who must carefully evaluate the quality and viability of the mastectomy flaps. This intraoperative decision-making process is critical, as flap thickness, perfusion, and tension influence the risk of flap ischemia and necrosis. In our cohort, flap necrosis was observed in 4 patients within the ADM group and only one patient required surgical debridement. This corresponds to a surgical revision rate for necrosis of only 1.6% in the ADM group. In contrast, the polyurethane group reported one case of flap necrosis (1,6%) that led to implant exposure and explantation.

Prepectoral reconstruction with smooth silicone implants may be performed without ADM in selected patients with robust flaps, but risks include higher pocket shear and malposition in borderline envelopes. ADM-assisted reconstructions provide pocket control and capsular modulation at the cost of more fluid-related events in some series. Polyurethane-covered devices achieve pocket stability through early tissue adhesion, which, consistent with our findings, may reduce fluid collections and reinterventions while maintaining similar patient-reported outcomes and capsular contracture rates.

Mastectomy flap necrosis rates vary quite significantly in literature. Our results compare favorably with the present literature, where flap necrosis requiring surgical revision may vary from 21.2% [32] to 0% in carefully selected cohorts [33]. Such discrepancies may reflect differences in surgical technique and institutional experience and patient selection. High-volume breast units with extensive expertise in prepectoral reconstruction may contribute to lower complication rates through optimized flap handling and rigorous intraoperative evaluation.

Two cases of implant explantation were recorded in the ADM group. The first involved a hematoma that was not evacuated and subsequently evolved into an infected periprosthetic collection two months postoperatively. The second occurred in a young patient who developed a persistent periprosthetic fluid collection requiring repeated percutaneous aspirations. Implant infection occurred during adjuvant chemotherapy.

In the polyurethane group, we recorded implant explantation in one patient due to initial mastectomy flap necrosis that led to implant exposure and infection.

In the polyurethane group, periprosthetic fluid collection was registered in only one patient. Our data suggest that the so-called “Velcro-like effect” supports early adhesion to the surrounding tissues, effectively reducing the dead space where periprosthetic fluid collection or hematoma might accumulate.

However, the same “Velcro-like effect” of polyurethane implants appears to be more frequently associated to implant rippling. Although rippling was more frequently observed in the polyurethane group (64% vs. 47% in ADM), the difference did not reach statistical significance. Despite this trend, our data showed a significantly higher rate of minor revisional procedures, particularly fat grafting in the ADM group. An important factor to consider is the age difference between groups. Patients in the ADM cohort were significantly younger, and it is plausible that younger patients have higher cosmetic expectations and may be more likely to request or accept secondary refinements. While not quantifiable, these subjective preferences may have played a key role in shaping patient-reported outcomes and revision rates.

On the other hand, when an implant is fully wrapped, the biologic interface is at the ADM surface, not the implant shell. The ADM is rapidly revascularized and integrates with the surrounding tissues and the capsule forms on the outer ADM surface. Thus, “tissue ingrowth into the implant” is not required for stability. Instead, fixation sutures and ADM integration provide pocket control. In contrast, the polyurethane arm relies on early foam adhesion to stabilize the device without ADM.

In vivo studies and long-term clinical series describe early fibrovascular ingrowth at the polyurethane–tissue interface, followed by durable adhesion even as the foam layer thins, mitigating rotation of anatomically shaped implants and down-modulating capsular organization. Modern prepectoral series report low late capsular contracture (CC) rates with polyurethane devices (7–8%), while large ADM-assisted cohorts have also reported low rates (2%); our matched cohort observed low and statistically comparable CC between groups (Baker ≥III: 8% polyurethane vs 4% ADM, *p* = 0.59) and no increased risk of rotation.

Patient satisfaction, as measured by the BREAST-Q™, was similar across groups in most domains. However, a trend toward higher satisfaction with outcome was observed in the polyurethane group (*p* = 0.060). This domain highlights the patient’s overall opinion of their reconstructive journey, including the perioperative course. Patients in the ADM group, especially those who experienced periprosthetic fluid collections requiring aspiration, likely underwent more hospital visits, which may have negatively influenced their perception of the reconstruction, even in the absence of major complications.

Additionally, because implant volume was a matching variable (± 80 mL), the distributions were closely overlapping (335 mL ADM vs 330 mL PU). Within this narrow range, we did not observe a visible volume–complication trend on inspection of the matched pairs, and the sample size precluded powered subgroup testing by volume stratification.

Although not the focus of this study, the use of polyurethane implants may offer economic advantages, a factor increasingly relevant in resource-constrained environments [33, 34].

In addition, several historical and contemporary studies support the biocompatibility and long-term safety of polyurethane-coated implants [35]. Castel et al. reported excellent outcomes in long-term follow-up, with low capsular contracture rates and no increased risk of implant-related complications [20]. A systematic review by Duxbury and Harvey also found polyurethane implants to be effective in reducing contracture while maintaining a favorable safety profile [21]. Recent literature has shifted towards expanding the indications for polyurethane implants in prepectoral reconstruction. Lisa et al. conducted a large single-center study with 317 prepectoral reconstructions even in patients with prior breast irradiation, comorbidities, further supporting the safety of polyurethane implants in real-world clinical scenarios. Major complications occurred in only 6.3% of cases, while minor complications occurred in 27.8% of cases, such as rippling (14.5%) and capsular contracture (7.6%) [34]. In addition to current literature, our findings further support this paradigm shift, suggesting that proper clinical assessment may outweigh rigid preoperative exclusion criteria.

Our study presents several strengths. The matched cohort design minimized baseline imbalances and controlled for critical confounding variables, such as implant volume, previous surgery, and radiotherapy. A comprehensive evaluation of objective surgical outcomes, including complication rates, implant loss, and the number of revisional procedures, was paired with validated patient-reported measures through the BREAST-Q™, allowing for a robust assessment of both clinical effectiveness and patient satisfaction. The inclusion of short- and medium-term follow-up and appropriate statistical analysis further strengthens the reliability of the findings.

Nonetheless, some limitations must be acknowledged. The retrospective nature of the study introduces potential selection and reporting bias. The relatively small sample size may have limited the ability to detect differences between groups. Moreover, some variables, such as esthetic outcomes and capsular contracture grading, are subjective and operator-dependent. Additionally, the absence of homogenous long-term follow-up data in all patients precludes conclusions about delayed outcomes such as contracture progression. Finally, although the overall time frame was consistent, the annual ratio between the use of ADMs and polyurethane implants slightly varied over the study period, potentially influencing follow-up distribution and the timing of observed outcomes.

Future research should aim to validate these findings through prospective, multicenter trials with larger patient cohorts and extended follow-up periods. In particular, studies should focus on long-term outcomes such as capsular contracture, implant stability, esthetic refinements, and patient satisfaction. Cost-effectiveness analyses will also be essential, especially given the rising demand for resource optimization and personalized care in breast reconstruction.

## Conclusion

This matched cohort analysis offers insight specific to immediate prepectoral reconstruction. Although both strategies remain valid options, micro-polyurethane implants are associated with a significantly lower incidence of periprosthetic fluid accumulation and subsequent percutaneous aspiration compared to ADM-assisted reconstructions.

## References

[1] Nava MB, Catanuto G, Pennati A, Garganese G, Spano A. Conservative mastectomies. Aesthet Plast Surg. 2009;33(5):681–6. 10.1007/s00266-009-9382-4. 10.1007/s00266-009-9382-4 19588190

[2] Amro C, Sorenson TJ, Boyd CJ, Hemal K, Vernice NA, Park JJ, et al. The evolution of implant-based breast reconstruction: innovations, trends, and future directions. J Clin Med. 2024;13(23):7407. 10.3390/jcm13237407. 39685866 10.3390/jcm13237407 PMC11642416

[3] Rancati A, Soderini A, Dorr J, Gercovich G, Tessari L, Gonzalez E. One-step breast reconstruction with polyurethane-covered implants after skin-sparing mastectomy. J Plast Reconstr Aesthet Surg. 2013;66(12):1671–5. 10.1016/j.bjps.2013.07.005. 23932524 10.1016/j.bjps.2013.07.005

[4] Scardina L, Di Leone A, Sanchez AM, Accetta C, Barone Adesi L, Biondi E, et al. Oncological safety of prepectoral implant-based breast reconstruction after conservative mastectomy: insights from 842 consecutive breast cancer patients. Cancers (Basel). 2025;17(6):925. 10.3390/cancers17060925. 40149261 10.3390/cancers17060925 PMC11939890

[5] de Vita R, Buccheri EM, Villanucci A, Pozzi M. Breast reconstruction actualized in nipple-sparing mastectomy and direct-to-implant, prepectoral polyurethane positioning: early experience and preliminary results. Clin Breast Cancer. 2019;19(2):e358–63. 10.1016/j.clbc.2018.12.015. 30691930 10.1016/j.clbc.2018.12.015

[6] Kaplan J, Wagner RD, Braun TL, Chu C, Winocour SJ. Prepectoral breast reconstruction. Semin Plast Surg. 2019;33(4):236–9. 10.1055/s-0039-1696966. 31632206 10.1055/s-0039-1696966 PMC6797493

[7] Scardina L, Di Leone A, Biondi E, Carnassale B, Sanchez AM, D’Archi S, et al. Prepectoral vs. submuscular immediate breast reconstruction in patients undergoing mastectomy after neoadjuvant chemotherapy: our early experience. J Pers Med. 2022;12(9):1533. 10.3390/jpm12091533. 36143318 10.3390/jpm12091533 PMC9504024

[8] Franceschini G, Scardina L, Di Leone A, Terribile DA, Sanchez AM, Magno S, et al. Immediate prosthetic breast reconstruction after nipple-sparing mastectomy: traditional subpectoral technique versus direct-to-implant prepectoral reconstruction without acellular dermal matrix. J Pers Med. 2021;11:153. 10.3390/jpm11020153. 33671712 10.3390/jpm11020153 PMC7926428

[9] Scarabosio A, ContessiNegrini F, Pisano G, Beorchia Y, Castriotta L, De Francesco F, et al. Prepectoral direct-to-implant one-stage reconstruction with ADMs: safety and outcome in “thin patients.” Clin Breast Cancer. 2023;23(8):e507–14. 10.1016/j.clbc.2023.08.007. 37735018 10.1016/j.clbc.2023.08.007

[10] Kaplan HY, Rysin R. Use of 801 acellular dermal matrices (ADMs) in direct-to-implant breast reconstruction: a clinical observation of complication profiles over a 7-year period. Aesthet Surg J. 2025;45(3):276–85. 10.1093/asj/sjae238. 39661417 10.1093/asj/sjae238

[11] Tork S, Jefferson RC, Janis JE. Acellular dermal matrices: applications in plastic surgery. Semin Plast Surg. 2019;33(3):173–84. 10.1055/s-0039-1693019. 31384233 10.1055/s-0039-1693019 PMC6680075

[12] Sbitany H, Mukhatyar V, Hammer J, Hoonjan A, Leung BK, Gardocki-Sandor M. Biologic response with and without acellular dermal matrix in breast reconstruction. Plast Reconstr Surg - Global Open. 2025;13(4):e6671. 10.1097/GOX.0000000000006671. 10.1097/GOX.0000000000006671 PMC11964387 40182297

[13] Liang R, Pan R, He L, Dai Y, Jiang Y, He S, et al. Decellularized extracellular matrices for skin wound treatment. Materials. 2025;18(12):2752. 10.3390/ma18122752. 40572885 10.3390/ma18122752 PMC12194566

[14] Siliprandi M, Vaccari S, Lisa AVE, Furlan S, Di Giuli R, Klinger FM, et al. Polyurethane-coated silicone breast implants: a viable option for primary augmentation mammoplasty: a systematic review and technical considerations. JPRAS Open. 2025;44:463–78. 10.1016/j.jpra.2025.03.019. 40452890 10.1016/j.jpra.2025.03.019 PMC12123325

[15] Correia-Pinto JM, Poleri F, Barbosa JP, Casimiro R, Azevedo MS, Andresen C, et al. Comparing polyurethane and acellular dermal matrix implant cover in prepectoral breast reconstruction: short-term complications. Plast Reconstr Surg Glob Open. 2023;11(2):e4798. 10.1097/GOX.0000000000004798. 36751508 10.1097/GOX.0000000000004798 PMC9894346

[16] Handel N, Gutierrez J. Long-term safety and efficacy of polyurethane foam-covered breast implants. Aesthet Surg J. 2006;26(3):265–74. 10.1016/j.asj.2006.04.001. 19338905 10.1016/j.asj.2006.04.001

[17] Scarpa C, Borso GF, Vindigni V, Bassetto F. Polyurethane foam-covered breast implants: A justified choice? Eur Rev Med Pharmacol Sci. 2015;19(9):1600–6. 26004599

[18] Loreti A, Siri G, De Carli M, Fanelli B, Arelli F, Spallone D, et al. Immediate breast reconstruction after mastectomy with polyurethane implants versus textured implants: a retrospective study with focus on capsular contracture. Breast. 2020;54:127–32. 10.1016/j.breast.2020.09.009. 33010626 10.1016/j.breast.2020.09.009 PMC7529839

[19] Kaur R, Singh P, Tanwar S, Varshney G, Yadav S. Assessment of bio-based polyurethanes: perspective on applications and bio-degradation. Macromol. 2022;2(3):284–314. 10.3390/macromol2030019.

[20] Castel N, Soon-Sutton T, Deptula P, Flaherty A, Parsa FD. Polyurethane-coated breast implants revisited: a 30-year follow-up. Arch Plast Surg. 2015;42(2):186–93. 10.5999/aps.2015.42.2.186. 25798390 10.5999/aps.2015.42.2.186 PMC4366700

[21] Duxbury PJ, Harvey JR. Systematic review of the effectiveness of polyurethane-coated compared with textured silicone implants in breast surgery. J Plast Reconstr Aesthet Surg. 2016;69(4):452–60. 10.1016/j.bjps.2016.01.013. 26887685 10.1016/j.bjps.2016.01.013

[22] Salgarello M, Pagliara D, Barone Adesi L, Visconti G, Wild JB, Matey P. Direct to implant breast reconstruction with prepectoral micropolyurethane foam-coated implant: analysis of patient satisfaction. Clin Breast Cancer. 2021;21(4):e454–61. 10.1016/j.clbc.2021.01.015. 33627298 10.1016/j.clbc.2021.01.015

[23] Chatterjee A, Nahabedian MY, Gabriel A, Macarios D, Parekh M, Wang F, et al. Early assessment of post-surgical outcomes with pre-pectoral breast reconstruction: a literature review and meta-analysis. J Surg Oncol. 2018;117(6):1119–30. 10.1002/jso.24938. 29346711 10.1002/jso.24938

[24] Berna G, Cawthorn SJ, Papaccio G, Balestrieri N. Evaluation of a novel breast reconstruction technique using the Braxon acellular dermal matrix: a new muscle- -sparing breast reconstruction. ANZ J Surg. 2017;87:493–8. 25266930 10.1111/ans.12849

[25] Weichman KE, Wilson SC, Weinstein AL, Hazen A, Levine JP, Choi M, et al. The use of acellular dermal matrix in immediate two-stage tissue expander breast reconstruction. Plast Reconstr Surg. 2012;129(5):1049–58. 10.1097/PRS.0b013e31824a2acb. 22544088 10.1097/PRS.0b013e31824a2acb

[26] Srivastava V, Basu S, Shukla VK. Seroma formation after breast cancer surgery: what we have learned in the last two decades. J Breast Cancer. 2012;15(4):373–80. 10.4048/jbc.2012.15.4.373. 23346164 10.4048/jbc.2012.15.4.373 PMC3542843

[27] Correia-Pinto JM, Andresen C, Barbosa JP, Poleri F, Casimiro R, Gonçalves D, et al. Impact of polyurethane versus acellular dermal matrix coating on prepectoral reconstruction outcomes: interface does matter. J Plast Reconstr Aesthet Surg. 2024;91:15–23. 10.1016/j.bjps.2024.01.025. 38401273 10.1016/j.bjps.2024.01.025

[28] Caputo GG, Mura S, Albanese R, Zingaretti N, Pier Camillo P. Seroma formation in pre-pectoral implant-based ADM assisted breast reconstruction: a comprehensive review of current literature. Chirurgia (Bucur). 2021;116(2 Suppl):16–23. 33963692

[29] Salgarello M, Visconti G, Barone-Adesi L. Current trends in breast reconstruction. Minerva Surg. 2021;76(6):526–37. 10.23736/S2724-5691.21.08987-5. 34935321 10.23736/S2724-5691.21.08987-5

[30] Frame J, Kamel D, Olivan M, Cintra H. The in vivo pericapsular tissue response to modern polyurethane breast implants. Aesthet Plast Surg. 2015;39(5):713–23. 10.1007/s00266-015-0550-4. 10.1007/s00266-015-0550-4 26304599

[31] Masià J, iBAG Working Group. The largest multicentre data collection on prepectoral breast reconstruction: the iBAG study. J Surg Oncol. 2020;122(5):848–60. 10.1002/jso.26073. 32786089 10.1002/jso.26073 PMC7540676

[32] di Santanelli Pompeo F, Firmani G, Paolini G, Amorosi V, Briganti F, Sorotos M. Immediate prepectoral breast reconstruction using an ADM with smooth round implants: a prospective observational cohort study. J Plast Reconstr Aesthet Surg. 2023;80:56–65. 10.1016/j.bjps.2023.02.014. 36989882 10.1016/j.bjps.2023.02.014

[33] de Vita R, Villanucci A, Buccheri EM, Pozzi M. Extended clinical experience with nipple-sparing mastectomy and prepectoral polyurethane implant positioning (BRAND4P method). Clin Breast Cancer. 2022;22(5):e623–8. 10.1016/j.clbc.2022.03.005. 35437225 10.1016/j.clbc.2022.03.005

[34] Lisa A, Riccardi F, Alessandri-Bonetti M, et al. Outcomes, indications and predictive factors of complications in postmastectomy prepectoral breast reconstructions with polyurethane foam-coated implants. Breast. 2025. 10.1016/j.breast.2025.104520. 40695028 10.1016/j.breast.2025.104520 PMC12305310

[35] National Toxicology Program. Bioassay of 2,4-diaminotoluene for possible carcinogenicity. Natl Cancer Inst Carcinog Tech Rep Ser. 1979;162:1–139. 12799700

